# Micellization Behavior of Long-Chain Substituted Alkylguanidinium Surfactants

**DOI:** 10.3390/ijms17020223

**Published:** 2016-02-06

**Authors:** Roza Bouchal, Abdellah Hamel, Peter Hesemann, Martin In, Bénédicte Prelot, Jerzy Zajac

**Affiliations:** 1 Institut Charles Gerhardt, UMR-5253 CNRS-UM-ENSCM, Place Eugène Bataillon, F-34095 Montpellier cedex 5, France; roza.bouchal@etud.univ-montp2.fr (R.B.); ha_ab1@yahoo.fr (A.H.); benedicte.prelot@umontpellier.fr (B.P.); 2 Department of Chemistry, University Badji-Mokhtar, BP12 Annaba, Algeria; 3 Laboratoire Charles Coulomb, UMR 5221 CNRS-UM, Place Eugène Bataillon, F-34095 Montpellier cedex 5, France; martin.in@univ-montp2.fr

**Keywords:** alkylguanidinium chlorides, surfactants, Krafft temperature, surface tension, critical micelle concentration, standard enthalpy of micelle formation

## Abstract

Surface activity and micelle formation of alkylguanidinium chlorides containing 10, 12, 14 and 16 carbon atoms in the hydrophobic tail were studied by combining conductivity and surface tension measurements with isothermal titration calorimetry. The purity of the resulting surfactants, their temperatures of Cr→LC and LC→I transitions, as well as their propensity of forming birefringent phases, were assessed based on the results of ^1^H and ^13^C NMR, differential scanning calorimetry (DSC), and polarizing microscopy studies. Whenever possible, the resulting values of Krafft temperature (*T*_K_), critical micelle concentration (CMC), minimum surface tension above the CMC, chloride counter-ion binding to the micelle, and the standard enthalpy of micelle formation per mole of surfactant (Δ_mic_*H*°) were compared to those characterizing alkyltrimethylammonium chlorides or bromides with the same tail lengths. The value of *T*_K_ ranged between 292 and 314 K and increased strongly with the increase in the chain length of the hydrophobic tail. Micellization was described as both entropy and enthalpy-driven. Based on the direct calorimetry measurements, the general trends in the CMC with the temperature, hydrophobic tail length, and NaCl addition were found to be similar to those of other types of cationic surfactants. The particularly exothermic character of micellization was ascribed to the hydrogen-binding capacity of the guanidinium head-group.

## 1. Introduction

The self-aggregation of amphiphilic molecules and ions into such supramolecular objects as micelles, vesicles, and membranes [1] plays an important role in many fields like biology, pharmacy, and materials science [2,3]. The diversity of such uses has been a powerful driving force behind the design and preparation of new families of surface-active agents over the last several decades [2,4,5,6,7,8].

Surfactant-templated synthesis of mesoporous inorganic materials is a good example of how the efforts aimed at surfactant development and characterization have guided the progress in conception, design and synthesis of advanced materials with tunable porous architecture, particle size and morphology [9,10,11]. The present paper is primarily concerned with a special case of the synthesis of nanostructured silica phases using soft templating approaches. Together with the structure of silylated precursors, the use of tailor made surfactants can be an important parameter to access structured materials. In this context, long-chain substituted guanidinium halides were recently considered as a promising alternative to alkyltrimethylammonium bromides in the preparation of silica hybrid materials displaying a regular architecture on a mesoscopic scale length [12]. The guanidinium group is the basis of a rich supramolecular chemistry due to its ability to interact with various substances via ionic bonds and hydrogen bonding. In combination with sulfonate, carboxylate or phosphonate groups, it can produce a large variety of hydrogen-bonded supramolecular structures [13,14,15]. Moreover, it facilitates surface etching of MOF crystals of the HKUST-1 type [16]. Guanidinium salts are also strong denaturating agents for proteins, and the denaturation mechanism is due to the action of hydrogen bonding [17,18]. Therefore, in order to get a deeper understanding of the observed differences in the templating mechanism, a detailed study of the self-aggregation behavior of guanidinium salts against that of alkyltrimethylammonium homologues seems indispensable.

The self-assembly and surface-activity of long-chain guanidinium surfactants have rarely been studied in the literature [7,19]. Miyake *et al.* reported an intensive study on the self-assembling characteristics of dodecylguanidine hydrochloride based on the experimental results on the phase diagram, area occupied per surfactant unit at the aqueous solution-air interface, micellar aggregation number, as well as thermodynamic functions of micelle formation [7]. It was argued that the increased assembly formability, compared to the dodecyltrimethylammonium (DTAC) homologue, was caused mostly by hydrogen bonding among guanidinium groups via water molecules, thereby overcoming the repulsive force between the cationic charges. The guanidinium head-groups were postulated to be closely packed both at the solution–air and micelle–solution interface in the absence of added background electrolyte, and the addition of even a small amount of NaCl was responsible for the observed shape change of the micelle growing to form the string-like structure. The micellization was demonstrated to be an exothermic and entropy-driven phenomenon. The Krafft temperature of the surfactant was determined to be about 293 K, much higher than that of DTAC. Song *et al.* carried out the surface tension and conductivity measurements at 298 K for two series of guanidinium-type surfactants, mono-alkylguanidinium and *N*,*N*,*N*′-dimethylalkylguanidinium chlorides containing 8, 10 and 12 carbon atoms in the alkyl chain [19]. These cationic surfactants showed antimicrobial activity, and their efficiency as antimicrobial agents increased as the length of the hydrophobic tail was increased. The *N*,*N*,*N*′-dimethylalkylguanidinium surfactants reduced the surface tension of water to about 30–33 mN·m^−1^, whereas the minimum surface tensions ranging between 23 and 25 mN·m^−1^ were observed with the mono-alkylguanidinium counterparts. The values of the critical micelle concentration, CMC, and chloride counter-ion binding to the micelle, β, were found to be chain-length dependent, in accordance with the general trends documented for numerous ionic surfactants [2]. In the above-mentioned two papers [7,19], a very small degree of micelle dissociation was deduced from the β values being close to unity.

The aim of the present paper is to report the results of a systematic study of surface activity and self-assembling behavior for a series of mono-alkylguanidinium chlorides containing 10, 12, 14 and 16 carbon atoms in the alkyl chain. The main characteristics were inferred from the appropriate conductivity, surface tension and calorimetry measurements. The possible increase in the Krafft temperature (*T*_K_) above room temperature upon increasing the alkyl chain length was corroborated by the determination of the *T*_K_ values for the four cationic surfactants studied here. Since only isothermal titration calorimetry guaranteed a leak-proof and temperature-controlled measuring system, this was the principal technique used to investigate the effect of the alkyl chain length, temperature and salt addition on the micelle formation. It is a commonly accepted premise [2,3] that the behavior of ionic surfactants in aqueous solutions and at interfaces is an intricate outcome of various kinds of interactions involving surfactant hydrophobic tails, ionized head-groups, and counter-ions, as well as water molecules able to form hydrogen bonds among themselves and with other dissolved species. In this context, direct calorimetry studies of such interactions may provide greater insight into the mechanism of surfactant-assisted synthesis of porous materials via soft templating approaches, in which cationic surfactants are suitable structure directing agents.

In comparison with the paper by Miyake *et al.*, the C_10_, C_14_ and C_16_-members of the guanidinium series were additionally characterized based on the research strategy adopted here. To complete the work by Song *et al.*, more information was provided on the self-assembly behavior of C_10_ and C_12_-guanidinium chlorides, representing the common part of the two studies. Therefore, a more specific goal here was to focus on the micellization performance of such long-chain guanidinium-type cationic surfactants, based on which their adequacy and relevance as potential structure-directing agents in preparation of nanostructured silica materials can be judged.

## 2. Results and Discussion

Several methods have been reported in the literature for the synthesis of guanidinium salts [20], including desulfurization-addition sequences, the addition of amines to electrophiles such as carbodiimides, cyanamides or chloramidinium salts [21,22], and the utilization of guanylation reagents [23]. The synthesis route employed here made use of 1*H*-Pyrazol-1-carboxamidine hydrochloride as guanylating reagent [24]. This method allowed a nearly quantitative transformation of the starting material to the desired product under mild reaction conditions and afforded guanidinium salts in high purities and high yields [25].

The phase transition temperatures of the resulting guanidinium chlorides, as inferred from appropriate DSC measurements, are given in Table 1. It is necessary to emphasize that the DSC thermograms (not shown here) indicate the crystalline character of the guanidinium compounds; DGC directly forms an isotropic melt, whereas DDGC, TDGC and CGC exist in the form of liquid-crystalline mesophases. The phase transition temperatures have been taken from the first heating and cooling cycle, as the salts did not crystallize once molten. It can be seen that increasing the length of the alkyl tail results in a significant increase in the phase transition temperature.

**Table 1:** Phases transition temperatures and enthalpies, as taken from the first heating and cooling cycle.

Compound	*T* (°C) Cr→LC Δ*H* (kJ·mol^−1^)	*T* (°C) LC→I Δ*H* (kJ·mol^−1^)
DCG	Heating: 54.1 °C; −28.3 kJ·mol^−1^	–
DDGC	Heating: 65.2 °C; −34.7 kJ·mol^−1^	Heating: 113.5 °C; −0.78 kJ·mol^−1^ Cooling: 112.3 °C; 0.79 kJ·mol^−1^
TDGC	Heating: 72.2 °C; −22.9 kJ·mol^−1^	Heating: 169.2 °C; −0.37 kJ·mol^−1^ Cooling: 160.5 °C; 0.36 kJ·mol^−1^
CGC	Heating: 76.8 °C; −38.2 kJ·mol^−1^	Heating: 189.9 °C; −0.62 kJ·mol^−1^ Cooling: 185.2 °C; 0.47 kJ·mol^−1^

**Figure 1:**
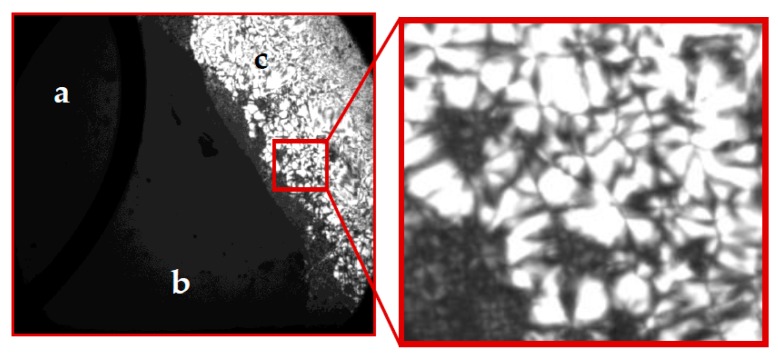
Polarizing micrographs of DGC at 2.5× original magnification: (**a**) air phase; (**b**) isotropic phase; (**c**) anisotropic phase.

The synthesized guanidinium chlorides are capable of forming birefringent phases in water, as can be seen in Figure 1. This behavior, although only evaluated qualitatively, indicates that guanidinium halides form anisotropic lyotropic phases. Additionally, the thermotropic behavior of these compounds was studied using differential scanning calorimetry. The formation of thermotropic mesophases is indicated by the C→LC and LC→I phase transitions of DDGC, TDGC and CGC (see Table 1). In this context, DGC has a particular position, since, although forming lyotropic solutions in aqueous medium, it does not form thermotropic phases, as shown by the absence of LC→I phase transition in its DSC thermogram.

### 2.1. Krafft Temperature, Critical Micelle Concentration, and Micelle Dissociation

**Figure 2:**
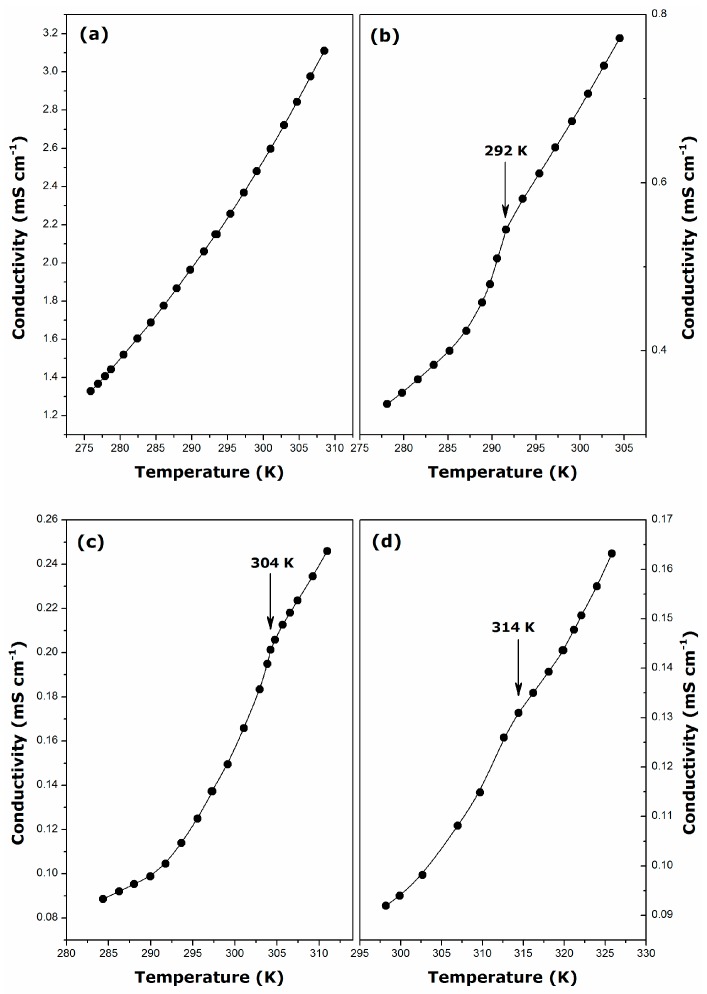
Plots of the specific conductance against temperature for micellar aqueous solutions of guanidinium cationic surfactants: (**a**) DGC (*m* = 31.72 mmol·kg^−1^); (**b**) DDGC (*m* = 8.01 mmol·kg^−1^); (**c**) TDGC (*m* = 2.55 mmol·kg^−1^); and (**d**) CGC (*m* = 0.7 mmol·kg^−1^).

Figure 2 illustrates the variations of the specific conductance of micellar aqueous solutions as a function of the temperature for the guanidinium surfactants studied in the present paper. With the sole exception of DGC, these plots exhibit a break, which indicates a noticeable change in the surfactant solubility and may be used to determine the Krafft temperature, *T*_K_ [26,27]. The following values have been inferred from the temperature dependency of the specific conductance: 292 ± 1 K (19 ± 1 °C), DDGC; 304 ± 1 K (31 ± 1 °C), TDGC; 314 ± 1 K (41 ± 1 °C), CGC. For DDGC, the Krafft temperature is, within the margin of error, in a good agreement with the value reported by Miyake *et al.* [7].

Since no such break has been observed in Figure 2a, the Krafft temperature of DGC should be lower than 276 K (3 °C), *i.e.* the lowest temperature to be obtained in the measuring system used. As for classical ionic surfactants [2], the Krafft temperature increases with the increase of the length of the alkyl chain. Compared to their alkyltrimethylammonium bromide or chloride homologues, the guanidinium type cationics appear to have much higher *T*_K_ values (e.g., *T*_K_ = 293 K, CTAB; *T*_K_ = 284 K, CTAC [28]). This also means that aqueous solutions of alkylguanidinium surfactants are more difficult to handle, especially in the case of CGC, where solvent evaporation may pose a serious problem.

**Figure 3:**
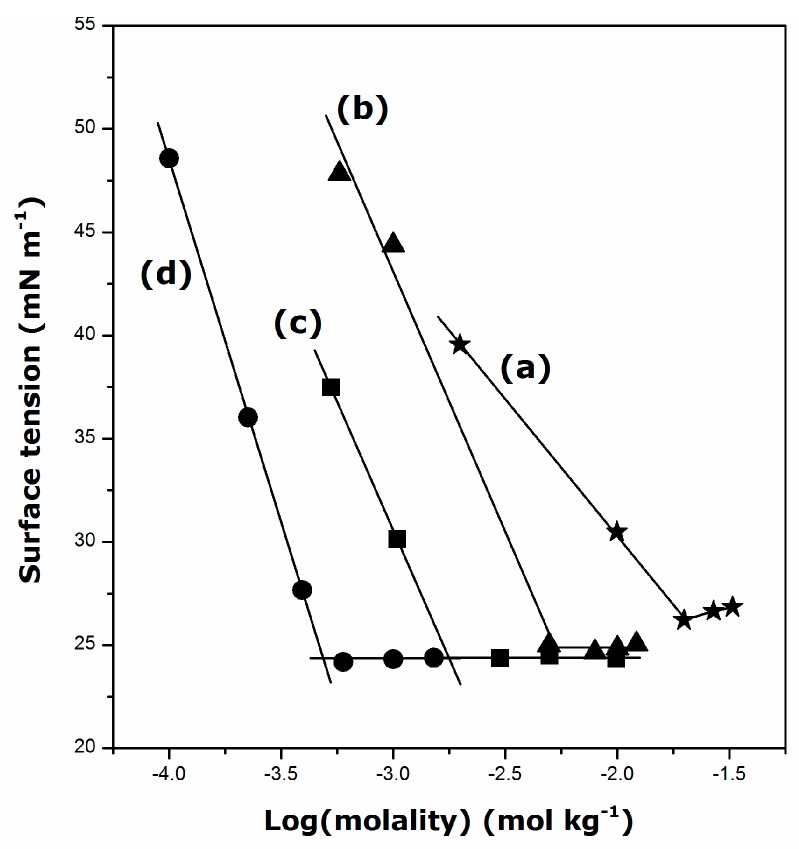
Plots of the surface tension against logarithm of the bulk molality for aqueous solutions of guanidinium cationic surfactants: (a) DGC at 298 K; (b) DDGC at 298 K; (c) TDGC at 306 K; and (d) CGC at 317 K.

In the next series of experiments, surface tension of surfactant solutions was determined at a constant temperature higher than the corresponding *T*_K_ value. The results are presented in Figure 3. The appearance of a shallow minimum close to the CMC, especially for DGC, might be interpreted as being the result of some surface-active impurities in the solutions, although the uncertainty of the surface tension measurements should not be forgotten here. Note that the high purity of the surfactant samples was confirmed by the NMR and FT-IR studies.

When analyzing Figure 3, the emphasis should be placed on the evaluation of the surfactant efficiency in lowering the surface tension of water. For example, DDGC decreases the surface tension down to about 24 ± 2 mN·m^−1^ above the CMC at 298 K, whereas dodecyltrimetylammonium chloride (DTAC) shows much lower efficiency (*i.e*., 42 mN·m^−1^ at 298 K [29]). This result is in agreement with those reported by other authors [7,19]. The propensity of guanidinium head-group to form hydrogen bonds may be considered to account for this reinforced surface activity of guanidinium cationics compared with their alkyltrimethylammonium homologues. The collective action of intermolecular hydrogen bonds makes water a highly structured liquid even at room temperature [3]. When the surfactant units are adsorbed at the water-air interface in an oriented fashion, new hydrogen bonds between water molecules and guanidinium head-groups may form easily, thereby preventing water molecules from binding as tightly to one another and thus lowering the tension of the surface. In the case of TDGC and CGC, the temperature dependence of the surface tension should be additionally taken into account, since water loses some of its peculiar structure properties at higher temperatures [30].

From plots of the surface tension *versus* the logarithm of a solution molality, it is also possible to evaluate the critical micelle concentration (CMC) or the area (*A*_min_) per adsorbed surfactant unit at the solution–air interface based on the Gibbs equation [2]. The following values have been obtained in the present work: CMC = 21 mmol·kg^−1^ and *A*_min_ = 1.4 nm^2^, DGC; CMC = 5.3 mmol·kg^−1^ and *A*_min_ = 0.8 nm^2^, DDGC; CMC = 1.8 mmol·kg^−1^ and *A*_min_ = 0.8 nm^2^, TDGC; CMC = 0.5 mmol·kg^−1^ and *A*_min_ = 0.6 nm^2^, CGC. In comparison with alkyltrimethylammonium chlorides containing the same alkyl chains and studied at the same temperature (*i.e.*, C_10_ and C_12_) [31], the guanidinium-type surfactants self-assemble at relatively lower CMC values. On the other hand, the alkylguanidinium cations occupy more surface area per molecule at the solution-air interface. Different shapes of the polar head-groups should be taken into account in rationalizing this last observation. Indeed, the ammonium group can be described as a nearly spherical bowl, thus leading to a C_3v_ symmetry of the long-chain-substituted ammonium surfactants [32]. On the contrary, the guanidinium group has a planar shape with a C_3h_ symmetry [33]. The present results show that the lengthening of the alkyl chain by two methylene groups causes a steady decrease in the *A*_min_ value, which thereby points out the increasingly enhanced adhesion of alkylguanidinium units within the adsorbed monolayer. This trend is at variance with the very high compactness of the surfactant monolayer already postulated by Miyake *et al.* for DDGC based on the *A*_min_ value of 0.37 nm^2^ [7]. Even though the small number of points in the molality region close to the break points in Figure 3 makes the resulting CMC and *A*_min_ values less precise here, there are no strong indications for unusually close packing of alkylguanidinium cations at the solution–air interface.

**Figure 4:**
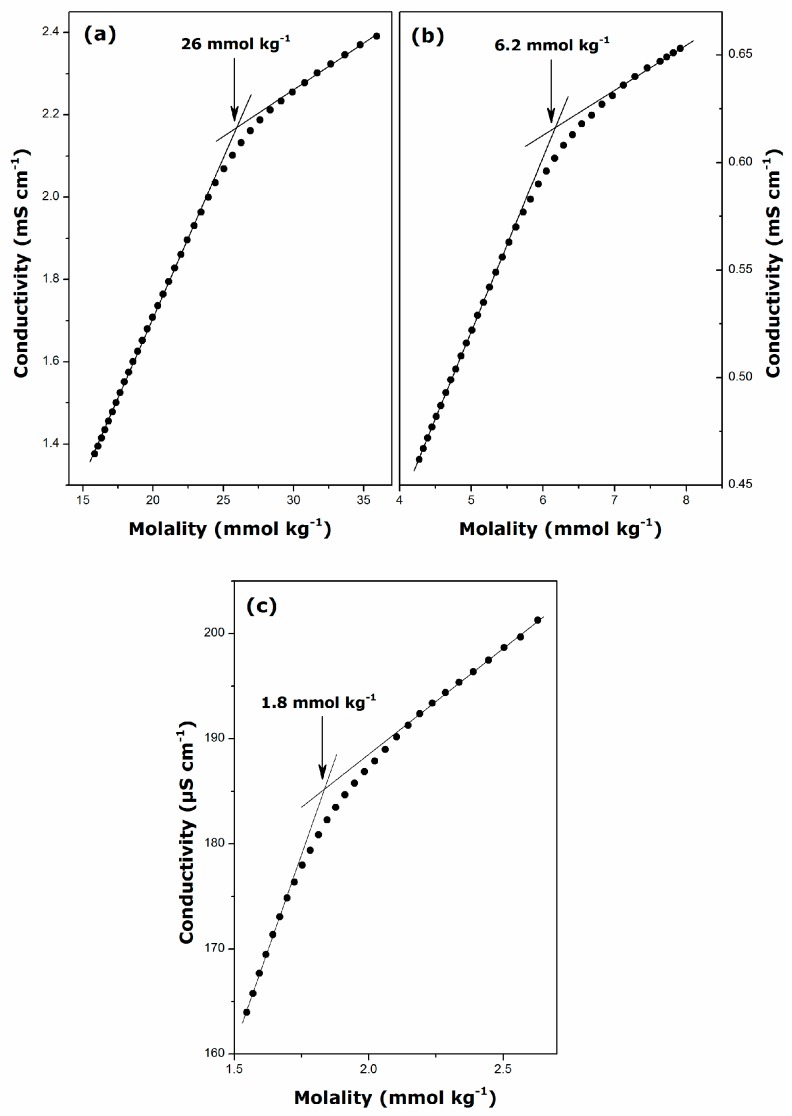
Plots of the specific conductance against bulk concentration for aqueous solutions of guanidinium cationic surfactants: (**a**) DGC at 298 K; (**b**) DDGC at 298 K; and (**c**) TDGC at 306 K.

More reliable values of micellization parameters for guanidinium surfactants have been inferred from the measurements of the specific conductance of aqueous solutions. For comparative purposes, the conductivity measurements were carried above the Krafft temperature: at 298 K, DGC and DDGC, and at 306 for TDGC. Taking into account the high Krafft temperature of the C_16_-homologue and given the difficulty in carrying out precise experiments at too high temperatures, further estimation of the main micellization parameters was restricted only to the first three members of the surfactant series. The conductivity *vs.* concentration plots are given in Figure 4 and the resulting values of CMC and counter-ion binding, β, to the micelle have been collected in Table 2.

**Table 2:** Critical micelle concentration, CMC, and degree of counter-ion binding to the micelle, β, for guanidinium cationic surfactants.

Surfactant	Temperature (K)	CMC (mmol·kg^−1^)	β	Δ_mic_*G*° (kJ·mol^−1^)
DGC	298	26 ± 1	0.71 ± 0.01	−22.3 ± 0.6
DDGC	298	6.2 ± 0.3	0.74 ± 0.01	−28.3 ± 0.9
TDGC	306	1.8 ± 0.1	0.72 ± 0.01	−33.6 ± 0.9

It should be noted here that the changes in the solution conductivity above the CMC observed in Figure 2 and Figure 4 are not very abrupt, which likely indicates that some partial Cl^−^ dissociation renders the micelles less effective charge carriers than the monomers. Of course, this hypothesis argues against the full counter-ion binding to the guanidinium micelles, at variance with the β values close to unity as reported previously [7,19]. The existence of globular micelles at smaller surfactant concentrations (still above the CMC), as revealed by the polarizing micrographs in Figure 1, additionally corroborates the conclusion with respect to the β parameters drawn in the present work. The comparison between alkylguanidinium cationics and their alkyltrimethylammonium homologues [7,34,35,36] indicates that the former form less ionized micelles. Miyake *et al.* have explained this increased counter-ion binding by a much closer packing of the DDGC units in micelles under the action of hydrogen bonds, thereby making the surface charge density higher than that of DTAC [7].

The generally used rule for ionic surfactants [2] that the CMC is divided by 4 on the addition of two methylene groups still holds approximately in this case. On the contrary, the β parameter seems to be much less sensitive to the alkyl chain length and temperature [34,35].

With the estimates of the two parameters, it was possible to determine the standard Gibbs free energy of micellization (Δ_mic_*G*°) of the surfactant under given conditions, using the following expression [2,37]:

Δ_mic_*G*° = R*T* (1 + β) ln *X*_CMC_ (1)

where *X*_CMC_ is the mole fraction of the surfactant in the aqueous solution at the CMC. In this formulation, Δ_mic_*G*° represents the Gibbs free energy of transfer of 1 mole of the surfactant solute from the aqueous phase to the micellar pseudo-phase. The resulting Δ_mic_*G*° values have been added to Table 2. As for all types of single-chain surfactants, the micellization of guanidinium cationics is a spontaneous phenomenon that is accompanied by a decrease in the Gibbs free energy. The Δ_mic_*G*° change involved in the transfer of a methylene unit of the hydrophobic group from an aqueous environment to the interior of the micelle at 298 K can be calculated from the two values obtained for DGC and DDGC. An increase in the length of the hydrophobic group makes Δ_mic_*G*° more negative by about 3.0 kJ per methylene group, which corresponds well to values usually reported for conventional surfactants [2,37,38].

### 2.2. Thermal Effects of Micelle Formation in Various Aqueous Media

**Figure 5:**
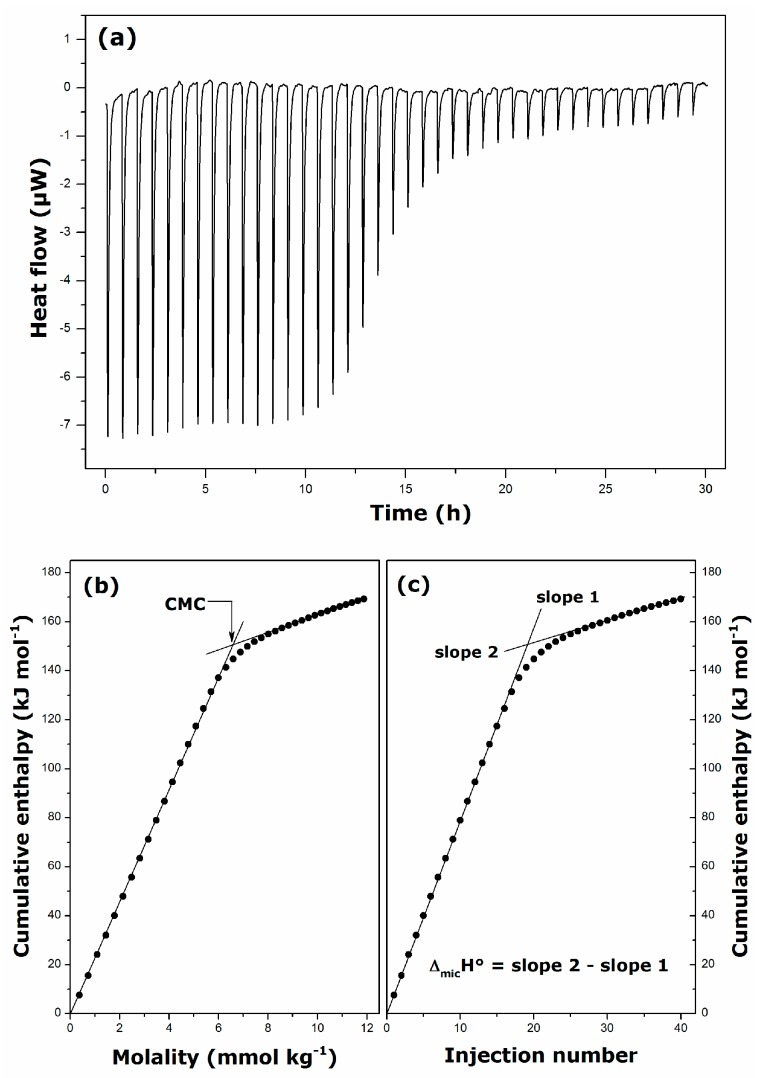
Results of calorimetry measurements of the cumulative enthalpy of dilution obtained by injecting a 60 mmol·kg^−1^ aqueous solution of DDGC into deionized water at 298 K: (**a**) records of 40 successive injections of 5 μL aliquots into a 1 mL glass ampoule containing initially 800 μL of deionized water (the equilibration time between 2 injections was set at 45 min); (**b**) cumulative enthalpy of dilution as a function of the equilibrium surfactant molality; (**c**) cumulative enthalpy of dilution as a function of the injection number.

Isothermal titration calorimetry (ITC) can provide useful information about the outcome of molecular interactions involved in the micellization phenomenon [39,40]. Given the high *T*_K_ temperatures of alkylguanidinium surfactants, the ITC equipment offers a better possibility of controlling the loss of water due to evaporation. An example of thermograms recorded during dilution calorimetry measurements is given in Figure 5a. The integration of peaks appearing in this thermogram leads to the determination of the total enthalpy changes during successive injections, Δ_inj_*H*_i_ [39,40]. The resulting thermal effects may be summed up to obtain the cumulative enthalpy of dilution per mole of the surfactant, Δ_dil_*H*_cum_, which can be expressed in the following manner [39,40]:

(2) $$\Delta_{\text{dil}}H_{\text{cum}}=\sum_{k=1}^{i}\frac{\Delta_{\text{inj}}H_{k}}{n_{2}^{\text{inj}}}=i\cdot \left[\Phi_{H}\left(m_{2}^{i}\right)-\Phi_{H}\left(m_{2}^{0}\right)\right]$$

where *i* is the number of successive injections, $n_{2}^{\text{inj}}$ is the number of moles of the surfactant solute (say component 2) injected into the calorimetric ampoule during each injection; $\Phi_{H}\left(m_{2}\right)$ represents the apparent molal enthalpy of the surfactant corresponding to a given molality $m_{2}$, $m_{2}^{0}$ is the molality of the stock solution. Taking into account the relationship between the apparent molal enthalpy, $\Phi_{H}\left(m_{2}\right)$, and the partial molal enthalpy, $\bar{h_{2}}\left(m_{2}\right)$, linear dependence of Δ_dil_*H*_cum_ against surfactant molality in Figure 5b indicates that $\bar{h_{2}}$ is a constant function of the molality in the pre-micellar and post-micellar regions [39]. Therefore, the intersection of the two linear portions provides estimate of the CMC for the surfactant in a given environment. In addition, the plot of Δ_dil_*H*_cum_ as a function of the injection number in Figure 5c is also represented as two straight lines intersecting at the CMC. In accordance with Equation (2), the standard enthalpy of micelle formation per mole of surfactant, Δ_mic_*H*°, is determined easily from the difference between the slopes of the two linear regression segments [39].

**Figure 6:**
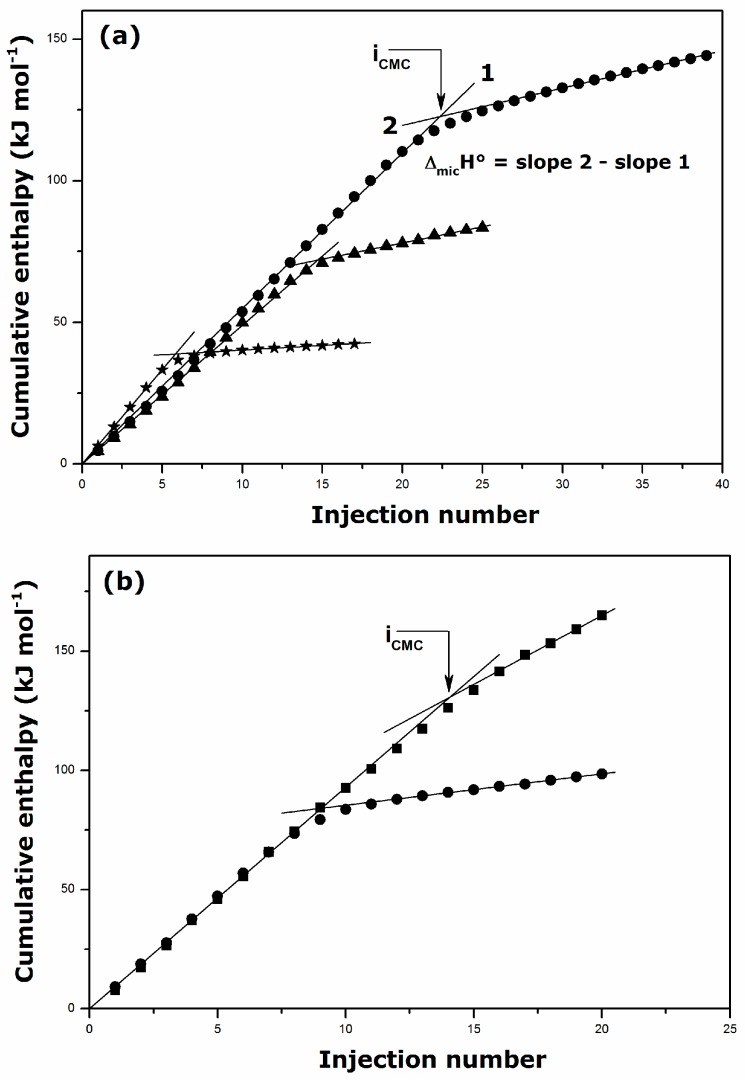
Effect of NaCl addition on the cumulative enthalpy of dilution per mole of DGC (**a**) and DDGC (**b**) determined by Isothermal Titration Calorimetry at 298 K: single-solute solution (circles), 0.001 NaCl solution (triangles), 0.01 NaCl solution (squares), 0.1 NaCl solution (stars). The enthalpy of dilution has been plotted as a function of the injection number to illustrate the direct calculation of the enthalpy of surfactant micellization.

Figure 6 shows that the linearity of the Δ_dil_*H*_cum_ *vs.* injection number plots is preserved for DGC and DDGC in presence of the background NaCl electrolyte. Similar curves were obtained for all alkylguanidinium and alkyltrimethylammonium surfactants studied here. The values of CMC and Δ_mic_*H*° were inferred accordingly from such data. These parameters have been collected in Table 3.

The discrepancies between the corresponding CMC values obtained in conductimetry and calorimetry experiments are obviously due to the differences in the sensitivity and reliability of the two methods and further data processing (e.g., the sharpness of the break in the concentration dependence of a given physical property [41]).

With the knowledge of such thermodynamic functions as Δ_mic_*G*° (Table 2) and Δ_mic_*H*° (Table 3), it is possible to calculate the standard molar entropy of micellization, Δ_mic_*S*°, for DGC, DDGC, and TDGC in single-component solutions. The following values have been obtained: *T*·Δ_mic_*S*° = 17.8 ± 0.9 kJ·mol^−1^, DGC at 298 K; *T*·Δ_mic_*S*° = 20.5 ± 2 kJ·mol^−1^, DDGC at 298 K; *T*·Δ_mic_*S*° = 16.1 ± 0.8 kJ·mol^−1^, TDGC at 306 K. When there is less than 14 carbon atoms in the alkyl chain, the negative values of Δ_mic_*G*° are mainly due to the positive values of Δ_mic_*S*°, Δ_mic_*H*°, even negative, being smaller than the value of *T*·Δ_mic_*S*°. As for other types of surfactants, micellization of guanidinium cationics is an entropy-driven process owing to the transfer of the hydrophobic tails from the water environment to the micelle core [3,42,43]. Therefore, the downward trends in the CMC with lengthening the alkyl chain, increasing the salt content, and decreasing the temperature, as observed qualitatively for DTAC, TTAC, DGC, and DDGC in Table 3, may be considered as consistent with the reinforcement of the hydrophobic effect in relation with changes in the peculiar solvent structure or in the micelle size and shape. In the case of TDGC, the enthalpy and entropy contributions to Δ_mic_*G*° are almost equal (but still opposite in sign) and the phenomenon also becomes enthalpy-driven. When analyzing the variations of Δ_mic_*H*° in Table 3, it should always be remembered that the thermal effect of micellization is a result of the interplay among various competitive or co-operative interactions; besides the hydrophobic interaction, the outcome of forces operating among the ionized head-groups, counter-ions and water molecules constitutes the most important contribution.

**Table 3:** Critical micelle concentration, CMC, and standard enthalpy of micelle formation per mole of the surfactant, Δ_mic_*H*°, for guanidinium cationic surfactants.

Surfactant	Solvent	Temperature (K)	CMC (mmol·kg^−1^)	Δ_mic_*H*° (kJ·mol^−1^)
DTAC	H_2_O	298	21.5 ± 0.1	5.1 ± 0.2
DTAC	0.01 M NaCl	298	18.1 ± 0.1	4.2 ± 0.1
DTAC	0.1 M NaCl	298	8.6 ± 0.1	3.6 ± 0.1
TTAC	H_2_O	298	5.0 ± 0.1	2.2 ± 0.2
TTAC	0.1 M NaCl	298	3.3 ± 0.4	1.1 ± 0.2
TTAC	H_2_O	318	6.0 ± 0.4	−8.9 ± 0.2
DGC	H_2_O	298	28.4 ± 0.1	−4.5 ± 0.1
DGC	0.001 M NaCl	298	25.5 ± 0.6	−4.2 ± 0.3
DGC	0.01 M NaCl	298	22.7 ± 0.2	−4.0 ± 0.1
DGC	0.1 M NaCl	298	9.6 ± 0.3	−6.4 ± 0.1
DDGC	H_2_O	298	6.2 ± 0.2	−7.8 ± 0.5
DDGC	0.001 M NaCl	298	5.7 ± 0.2	−9.0 ± 0.1
DDGC	0.01 M NaCl	298	3.5 ± 0.1	−9.5 ± 0.5
DDGC	H_2_O	306	6.6 ± 0.4	−12.3 ± 0.9
DDGC	H_2_O	310	7.3 ± 0.4	−13.0 ± 0.4
TDGC	H_2_O	306	1.5 ± 0.3	−17.5 ± 0.3

By far the most important conclusion drawn from the analysis of Table 3 is that the micellization of alkylguanidinium chlorides is an exothermic phenomenon, irrespective of the experimental conditions used. From this point of view, this micellization behavior is at variance with the endothermic micelle formation observed here for their alkyltrimethylammonium homologues. A more detailed comparison with the thermodynamic parameters reported in the literature for various ionic surfactant indicates that this behavior is rather similar to that exhibited by alkyltrimethylammonium bromides with the same hydrophobic tails, even though the micellization of the guanidinium cationics studied occurs at lower CMC values [44]. The latter conclusion may be easily rationalized since the degrees of counter-ion binding to the micelle are similar in both cases, and Cl^−^ is known to be more effective than Br^−^ in salting out the surfactant cation and thus depressing the CMC [2]. In accordance with some previously reported results [35,36], a passage from endothermic to exothermic micellization for alkyltrimethylammonium chlorides is favored with increasing the temperature and NaCl addition to the aqueous phase (see the results reported for DTAC and TTAC in Table 3). From the viewpoint of enthalpy, the present alkylguanidinium surfactants thus behave as their alkyltrimethylammonium homologues at higher temperatures and salt contents. This clearly points towards the guanidinium head-group as being responsible for the particularly exothermic character of micellization.

When the DDGC units self-assemble to form micelles in the presence of background electrolyte, only the first addition of NaCl to the aqueous phase makes Δ_mic_*H*° much less negative, and the thermal effect then seems to level off. This likely means that the DDGC micelles are fairly compact already in single-component solutions, but not to the degree proposed by Miyake *et al.* [7]. They then attain their optimal shape and size (e.g., maximum aggregation number) at low ionic strengths. For the same tail lengths, the fractions of surfactant units in the micellized state should be thus much greater than those characterizing the cationic micelles of alkyltrimethylammonium chlorides. For DGC in the presence of NaCl content up to 0.01 mmol·kg^−1^, a small decrease in the CMC is paralleled by a near constant value of Δ_mic_*H*°. Here, the salt addition to the aqueous phase does not cause much change in the self-aggregation behavior of the guanidinium type surfactants. High NaCl contents (of the order of 0.1 mmol·kg^−1^) render the phenomenon again more exothermic with the concomitant decrease in the CMC. Since the pre-micellar and post-micellar parts of the Δ_dil_*H*_cum_ *vs.* injection number plots preserve their linear character (see Figure 6a), it may be argued that the DGC micelles formed at high ionic strengths have a different shape from what is observed in single-solute solutions or at lower NaCl contents.

A stronger counter-ion binding in the Stern layer and hydrogen bonding with water molecules are also to be taken into account. Stronger interactions in the polar mantle of the micelle likely account for the enthalpy gain when the cationic head-groups are transferred from the aqueous environment to the micelle mantle. The increased magnitude of the exothermic micellization for longer hydrophobic tails may still be explained on the same basis when postulating the concomitant increase in the micelle aggregation number.

## 3. Materials and Methods

### 3.1. Chemicals

ACS reagent acetone and absolute methanol were purchased from Sigma-Aldrich (St Quentin Fallavier, France). Cationic surfactants used for comparative purposes, dodecyltrimethylammonium chloride (DTAC) and tetradecyltrimethylammonium chloride (TTAC), were also the products of Sigma-Aldrich with a high purity (purum, ≥98.0%). ACS reagent 1*H*-Pyrazol-1-carboxamidine hydrochloride and primary amines, decylamine, dodecylamine, tetradecylamine, and hexadecylamine were obtained from ABCR (Karlsruhe, Germany). All chemicals were employed without further purification. The 18.2 MΩ-cm water utilized in calorimetry, conductivity, and surface tension measurements was obtained with the aid of a combined Purite Select Analyst (France Eau, Fargues St Hilaire, France) and PURELAB^®^ Classic (ELGA LabWater, Atony, France) water purification system.

### 3.2. Synthesis and Characterization of the Guanidinium Surfactants

^1^H and ^13^C spectra in solution were recorded on Bruker AC 250 or Bruker Avance 400 spectrometers (Bruker, Rheinstetten, Germany) at room temperature. Deuterated chloroform and deuterated methanol were used as solvent for liquid NMR experiments, and chemical shifts are reported as δ values in parts per million relative to tetramethylsilane. FT-IR spectra were measured on a Perkin-Elmer 1000 FT-IR spectrometer (Perkin Elmer, Waltham, MA, USA); the samples were prepared as KBr pellets.

Decyl-, dodecyl-, tetradecyl- and hexadecylguanidinium chlorides were obtained as crystalline solids in high yield from the corresponding primary alkylamines by reaction with 1*H*-pyrazole-1-carboxamidine hydrochloride following the procedure published by Sasaki *et al.* [25]. In a general procedure, the monoalkyl-guanidinium salts were synthesized as follows. The primary amine (5.5 mmol) and 1*H*-Pyrazole-1-carboxamidine hydrochloride (5 mmol, 0.73 g) were dissolved in 10 mL of methanol. The resulting homogeneous solution was stirred during 20 h at 313 K. After this time, the solvent was evaporated and the formed pyrazole was eliminated by sublimation. The resulting guanidinium salt was purified by repeated recrystallization in acetone. After drying at 323 K under vacuum, the pure products were obtained as white powders in 80%–90% yield. The purity of the monoalkyl-guanidinium salts was checked via liquid NMR spectroscopy, as specified below.

#### 3.2.1. Decylguanidinium chloride (DGC)

^1^H NMR (CDCl_3_): δ = 0.81 (3H, “t”, *J* = 9.6 Hz); 1.19 (14H, m); 1.54 (2H, m); 3.13 (2H, “q”); 6.94 (4H, bs); 7.62 (1H, “t”). ^13^C NMR (MeOH-*d*_4_): δ = 14.15; 22.72; 26.84; 28.78; 29.35; 29.40; 29.62; 29.68; 31.95; 41.88; 157.33.

#### 3.2.2. Dodecylguanidinium chloride (DDGC)

^1^H NMR (CDCl_3_): δ = 0.81 (3H, t, *J* = 6.8 Hz); 1.18 (16H, m); 1.23 (2H, m); 1.54 (2H, q); 3.09 (2H, m); 7.09 (4H, m); 7.67 (1H, s). ^13^C NMR (CDCl_3_): δ = 14.16; 22.74; 26.87; 28.81; 29.40; 29.45; 29.68; 29.76; 29.79; 31.98; 41.91; 157.36. FT-IR (neat) ν_max_/cm^−1^ 3391, 3307, 3133, 2952, 2919, 2850, 1670, 1639, 1607, 1557, 1478, 1464, 1385, 1135, 1035, 887, 695.

#### 3.2.3. Tetradecylguanidinium chloride (TDGC)

^1^H NMR (CDCl_3_): δ = 0.90 (3H, “t”); 1.20–1.50 (22H, m); 1.61 (2H, m); 3.17 (2H, m); 7.10 (4H, m); 7.75 (1H, “t”). ^13^C NMR (CDCl_3_): δ =13.49; 22.76; 26.75; 28.93; 29.38; 29.50; 29.68; 29.79 (5 C); 32.10; 41.54; 157.64.

#### 3.2.4. Cetylguanidinium chloride (CGC)

^1^H NMR (CDCl_3_): δ = 0.91 (3H, “t”); 1.20–1.50 (26H, m); 1.66 (2H, m); 3.17 (2H, m); 7.21 (4H, m); 7.85 (1H, “t”). ^13^C NMR (CDCl_3_): δ = 13.02; 22.32; 26.31; 28.50; 28.93; 29.06; 29.25; 29.28; 29.35; 29.37 (5 C); 31.66; 41.09; 157.13.

Furthermore, all mono-alkylguanidinium cations were identified via high-resolution mass spectroscopy (HR-MS) making use of Waters Q-Tof mass spectrometer (Waters, Milford, MA, USA). Table 4 gives a comparison between the calculated and experimentally obtained *m*/*z* values.

**Table 4:** Results of high-resolution mass spectroscopy experiments with the guanidinium surfactants studied.

	Surfactants			
	DGC [C_11_H_26_N_3_]^+^	DDGC [C_13_H_30_N_3_]^+^	TDGC [C_15_H_34_N_3_]^+^	CGC [C_17_H_38_N_3_]^+^
(ESI+, *m*/*z*) [M]+ calc.	200.2121	228.2434	256.2747	284.3060
(ESI+, *m*/*z*) [M]+ found	200.2127	228.2434	256.2756	284.3061

The phase transition temperatures were determined by differential scanning calorimetry (NETZSCH PSC 204 F1 Phoenix was equipped with dinitrogen cryostatic cooling, 5–15 mg samples, 2 K·min^−1^ heating and cooling rates), and calibrated using an indium primary standard. Optical characterization of the compounds and the detection of mesophases were performed with a polarizing microscope (Leitz 12 POL S, Leitz, Wetzlar, Germany), equipped with a 1024 pixel × 768 pixel Sony CCD camera and an Instec hot stage regulated at 0.1 °C. Powder samples were deposited between slides and cover slips and inserted into the hot stage at room temperature. The temperature ramp typically was of 1 °C·min^-1^. The phase transitions were visually detected from the texture changes observed between crossed polarizers. The phase transitions were similarly detected by cooling the samples from the isotropic phase.

### 3.3. Physical Measurements

The surfactant solutions were prepared on a *molality* basis by taking the moles of solute dissolved in 1 kg of pure solvent.

#### 3.3.1. Conductimetry

The specific conductance of aqueous solutions was measured using a CDM210 conductivity meter (Radiometer Analytical, Villeurbanne, France) equipped with a 2-pole platinized XE100 cell. The calibration procedure was performed with a 0.1 mol·L^−1^ KCl standard solution (purchased from VWR Chemicals, Fontenay-sous-Bois, France) at 298 K (25 °C). The temperature was controlled using an external thermostat (LAUDA RE 206 Chiller/Heater Circulator, Lauda, Roissy-en-France, France) connected to a double-walled water container in which a measuring beaker was placed. The external water circulation loops were insulated, thus reducing the heat exchange with the environment. The solution was homogenized at a constant stirring rate with the aid of a small magnetic stirring bar. A TITRONIC^®^ (Schott Geräte, Mainz, Germany) piston burette was used for delivering precise doses of a given stock solution to the beaker. The main parts of the measuring and dosing systems were placed in a special thermostated cage in order to speed up the temperature stabilization inside the beaker. The actual temperature of the surfactant solution was measured using a T201 temperature sensor (±0.1 °C). At each concentration and temperature, the conductance reading was checked every 20 s until it reached a steady value. The repeatability of the conductivity measurements, estimated from two successive runs, was about ±0.5%.

The critical micelle concentration, CMC, and the degree of counter-ion binding to the micelle, β, were inferred from the plots of the specific conductance against the equilibrium surfactant concentration. For this purpose, each concentration series was made up by progressive injections of a micellar stock solution into the beaker containing initially the pure solvent. The resulting conductivity curves were represented as two linear regression segments having different slopes in the pre-micellar and post-micellar regions and meeting at the breakpoint: The CMC value was determined from the abscissa of the breakpoint, whereas the ratio of the two slopes allowed the evaluation of the β parameter. The relative uncertainties in the CMC and β, related to the precision of the conductivity measurements and further data processing, were less than ±5% and ±1%, respectively.

For the Krafft temperature measurements, the conductivity of a micellar solution was measured as a function of the temperature. The beaker filled with such a solution was cooled down first in a refrigerator at 276 K (3 °C) overnight and then inside the double-walled water container. The temperature of the solution was slowly increased under constant stirring in 1–2 degree steps at intervals of 30 min and its specific conductivity monitored by the same measuring system. The Krafft temperature, *T*_K_, was taken at the first distinctive break in the plot of conductivity *vs.* temperature. The values of *T*_K_ were estimated to be reproducible within 1 K.

#### 3.3.2. Surface Tension Measurements

The Wilhelmy plate method was employed to measure the equilibrium surface tension of surfactant solutions, making use of a Krüss digital tensiometer K 12 equipped with a platinum plate. The plate was cleaned thoroughly in a burner flame before the first measurement, and was then washed with acetone and carefully dried after each series of measurements. For each surfactant, 5–6 solutions at given concentrations in the submicellar and supermicellar ranges were prepared separately. Special attention has been paid to the temperature stabilization of the solution in the measuring vessel in the tensiometer chamber (20 min). The reliability of the measurement was assessed to be the best for the surfactant solutions above the CMC; here, the repeatability was slightly better than 10%.

#### 3.3.3. Titration Calorimetry

The enthalpy changes accompanying the dilution of a surfactant stock solution were monitored by means of a differential TAM III microcalorimeter operating in a heat flow mode. A high precision liquid thermostat (oil heat exchanger with Peltier coolers) maintained the temperature constant within ±0.0001 degrees. The experimental setup was equipped with a computer-controlled micro-syringe injection device allowing small amounts of a micellar stock solution to be injected into a 1 mL glass ampoule, in which a given mass of solvent was initially placed. The same volume of solvent was put in the reference cell. Then, both cells were returned to the microcalorimeter and the thermal equilibrium was reached after 2–4 h. Successive injections of the 5- or 10-μL aliquots of a given stock solution during 10 s resulted in an electric signal directly fed into a computer; the digitized signal representing the related thermal peaks was recorded with an equilibration time of 45 min applied between 2 injections. The homogeneity of the solution inside the measuring calorimetric cell was maintained by means of an agitation system including a gold stirrer rotated at a slow speed of 90 rpm. Integration of the areas under the thermal peaks was performed, and the resulting enthalpy of dilution Δ_inj_*H*_i_ was related to the amount of surfactant injected into the cell. The molality of each stock solution was chosen to be approximately 10 times the CMC of the surfactant at a given temperature and salt content. The repeatability of the calorimetry measurements was checked thoroughly by carrying out two simultaneous experiments with the use of two microcalorimeters working under the same conditions.

## 4. Conclusions

With their Griffin’s hydrophilic-lipophilic balance (HLB) ranging between 4 and 6, straight-chain guanidinium cationics DGC, DDGC, TDGC, CGC are less soluble in water and more surface-active than alkyltrimethylammonium bromides or chlorides having the same hydrophobic tails. These conclusions are corroborated by the following trends inferred from the experimental studies undertaken within the framework of the present work:

- For DDGC, TDGC, CGC, the Krafft temperature ranges between 292 and 314 K, and it increases strongly with the addition of methylene units to the surfactant hydrophobic tail.
- The presence of DDGC, TDGC, and CGC units in the aqueous solution decreases its surface tension down to about 24 mN·m^−1^ above the CMC, irrespective of the surfactant structure and temperature.
- The binding of chloride counter-ions to the micelle is about 70%, similar to that in the micelles of alkyltrimethylammonium bromides and much greater than in the case of alkyltrimethylammonium chlorides; it appears hardly dependent on the tail length and temperature.
- Micellization of guanidinium cationics, especially those with long alkyl chains, may be described as both entropy and enthalpy-driven; the critical micelle concentration, CMC, decreases and the standard enthalpy of micellization per mole of surfactant, Δ_mic_*H*°, becomes more negative (the process is more exothermic) upon lengthening the hydrocarbon tail.
- The addition of the background NaCl electrolyte to the aqueous phase causes a steady decrease in the CMC and renders the micellization process more exothermic; depending on the tail length, the alkylguanidinium micelles may attain their optimum shape and size at a given NaCl content, and further salt addition may result in the formation of micelles likely differing in shape and size.

Compared to alkyltrimethylammonium chlorides, the increased surface activity of alkylguanidinium surfactants, and the fact that micelle formation is much more exothermic and occurs at much lower CMC values, may be ascribed to the hydrogen-binding capacity of the guanidinium head-group, which promotes the formation of more compact micelles together with a stronger Cl^−^ binding in the corresponding Stern layer.
